# Craniopharyngioma: Survivin expression and ultrastructure

**DOI:** 10.3892/ol.2014.2690

**Published:** 2014-11-07

**Authors:** JIANG ZHU, CHAO YOU

**Affiliations:** 1 Department of Neurosurgery, West China Hospital, Sichuan University, Chengdu, Sichuan, P.R. China; 2 Department of Neurosurgery, Sichuan Cancer Hospital, Chengdu, Sichuan, P.R. China

**Keywords:** craniopharyngioma, survivin, expression

## Abstract

The aim of the present study was to investigate the significance of survivin protein expression levels in craniopharyngioma. Tumor samples and clinical data were obtained from 50 patients with craniopharyngioma who were admitted to the West China Hospital of Sichuan University (Chengdu, China). The morphology of the craniopharyngioma samples was observed using optical and electron microscopes, and survivin expression was investigated in the samples by immunohistochemical analysis. The immunohistochemical results revealed survivin expression in all of the craniopharyngioma samples, but not in the healthy brain tissue samples. It was identified that survivin was expressed at a higher level in cases of the adamantinomatous type compared with those of the squamous-papillary type, in male patients compared with female patients, in children compared with adults and in recurrent cases compared with non-recurrent cases. Furthermore, no significant difference was detected in survivin expression levels among the tumors of different subtypes and different disease stages. The results of the present study indicate that survivin is significant in the development of craniopharyngioma, and that survivin protein expression levels are a meaningful indicator for assessing craniopharyngioma recurrence.

## Introduction

Craniopharyngioma is a slow-growing benign tumor that develops in the parasellar or sellar region of the brain (1,2). Accounting for 6–9% of brain tumors in children (3), craniopharyngioma is the third most common type of childhood intracranial tumor (4) and the most common type of pediatric tumor in the hypothalamus and pituitary region, worldwide (5). In adults, craniopharyngioma typically occurs after the age of 60 years. According to an American study, the incidence rate of this tumor is low, at ~0.13 cases per 100,000 individuals/year, and does not vary with gender or ethnicity (6). Although craniopharyngioma can be highly aggressive and has a tendency to recur following surgical removal, the literature indicates that it rarely demonstrates malignant behavior (7–9). Despite the tumor being histologically benign, it is connected to surrounding vital structures, such as the pituitary gland, the optic chiasm, the circle of Willis and the hypothalamus, creating challenges for craniopharyngioma treatment and a high rate of morbidity (10). As demonstrated by data provided by the National Cancer Database, the five-year survival rate of patients with these tumors is 80%, and this rate decreases with increasing age (6).

Despite continuous progress in microsurgery techniques and years of research, the treatment of craniopharyngioma remains a significant problem, particularly for children (11). The unique anatomical structure of the suprasellar region and the connection of the tumor to surrounding tissues with critical functions results in difficulties in the removal of the tumor (3,12). With the development of novel surgical techniques and post-operative endocrine therapy, mortality and morbidity caused by craniopharyngioma have been greatly reduced, with the rate of total resection reaching up to 90% (12,13). However, a certain degree of controversy remains regarding the origin, histology and pathology of craniopharyngioma, as well as the optimal treatment strategy (2,14,15).

In the present study, the ultrastructure of craniopharyngioma was observed using electron microscopy and immunohistochemistry, with the aid of digital image analysis techniques, and survivin protein expression levels in craniopharyngioma were assessed to determine the biological characteristics and pathogenesis of craniopharyngioma.

## Materials and methods

### Patients and inclusion criteria

The paraffin-embedded samples of 50 patients admitted to the West China Hospital of Sichuan University (Chengdu, China) and diagnosed with craniopharyngioma following surgical resection between January 2000 and December 2005 were selected for use in the present study. The inclusion criteria were as follows: i) A complete patient medical history must be available; ii) the patient must have undergone pre-operative imaging (computed tomography or magnetic resonance imaging); iii) the pathology sample must be a sufficient size and have been established as craniopharyngioma by the Department of Pathology of the West China Hospital; and iv) the patient must have not have received radiation therapy, so as to eliminate the impact of radiation. According to the standard classification of central nervous system tumors published by the World Health Organization in 2000 (16), 33/50 cases (66%) were adamantinomatous craniopharyngioma and 17/50 cases (34%) were squamous-papillary craniopharyngioma. The present study included 32 male and 18 female patients, aged 4–66 years old (mean age, 29.12 years old). All of the patients were followed up by phone until November 2010. A total of 10 healthy brain tissue samples obtained from epileptic patients that had undergone surgery were used as the control group. This study was approved by the ethics committee of West China Hospital of Sichuan University and written informed consent was obtained from all patients.

### Measuring survivin expression

Survivin protein expression levels in the craniopharyngioma tissues were detected using immunohistochemistry and then quantitatively analyzed. Apoptotic cells were stained with transferase dUTP nick end labeling-peroxidase (TUNEL-POD; Roche, Branchburg, NJ, USA) and observed using transmission electron microscopy (Hitachi H-600IV; Hitachi, Ltd., Tokyo, Japan). Under the light microscope, survivin-positive cells were observed as brown granules in the nucleus or cytoplasm. For each section, a charge-coupled device camera was used to capture at least five fields of view at a magnification of ×400 (Olympus BX51 CCD; P70; Olympus Corporation, Tokyo, Japan), and Image-Pro Plus software (version 5.0; Media Cybernetics, Rockville, MD, USA) was used to quantitatively analyze the images; integrated optical density (IOD) was selected as the parameter to indicate survivin protein expression levels and the average IOD of all the recorded images was determined. Furthermore, TUNEL-POD stained the nuclei of the apoptotic cells yellow or brown, while the nuclei of the non-apoptotic cells were blue.

### Statistical analysis

Statistical analysis was performed using SPSS software, version 13.0 (SPSS, Inc., Chicago, IL, USA). Non-parametric tests were performed for each factor, and multinomial and ordinal logistic regression was performed. P<0.05 was used to indicate a statistically significant difference.

## Results

### Morphological observation

**Figure 1:**
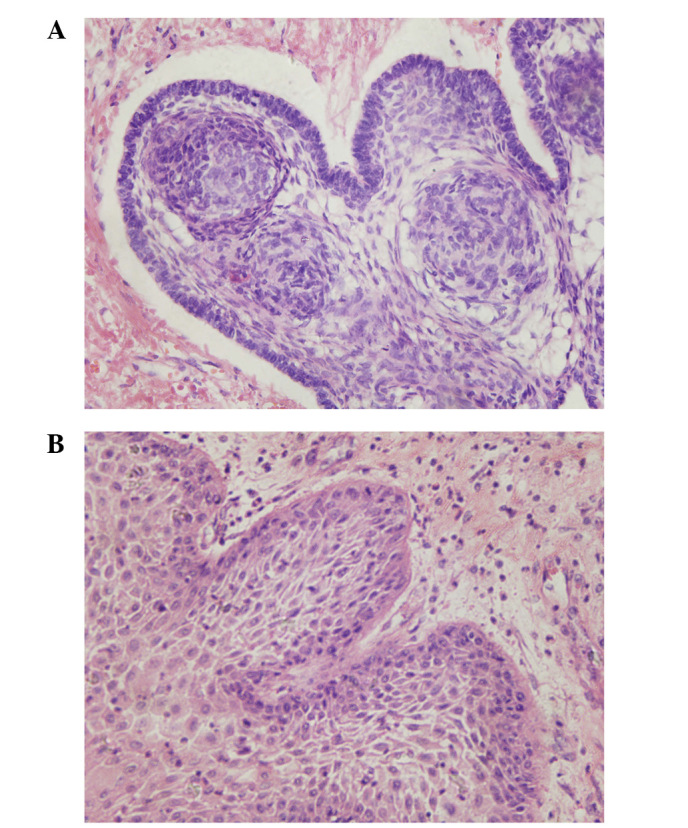
(A) Adamantinomatous and (B) squamous-papillary craniopharyngioma section images captured using light microscopy (stain, hematoxylin and eosin; magnification, ×400).

**Figure 2:**
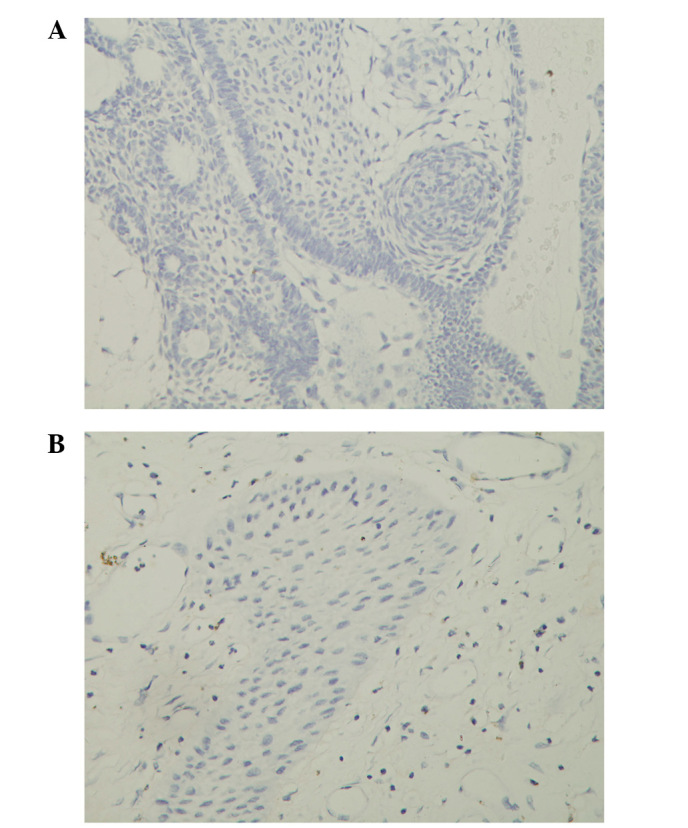
Transferase dUTP nick end labeling-peroxidase staining of craniopharyngioma sections demonstrating cell apoptosis in (A) adamantinomatous and (B) squamous-papillary craniopharyngioma tissue samples (magnification, ×400).

Adamantinomatous (Fig. 1A) and squamous-papillary (Fig. 1B) craniopharyngioma were observed using light microscopy following hematoxylin and eosin staining. Adamantinomatous craniopharyngioma is often observed in children and has cell cord and island similar to that of ameloblastoma. The observations revealed that the outermost layer of cells were palisade basal cells, which are cylindrical cubic epithelial cells, while the middle layer of cells were stratified polygonal squamous epithelial cells and the innermost layer of cells consisted of loosely structured reticular cells and wet keratin. Furthermore, local invasion of the surrounding healthy brain tissue was observed. By contrast, squamous-papillary craniopharyngioma is predominantly observed in adults, exhibiting a unique epithelium with a fibrovascular center. The present observations revealed that no microcapsules appeared to have formed, however, a palisade-like nucleus, a keratin pearl, wet keratin, calcification and significant inflammation were observed. The majority of cells in the adamantinomatous (Fig. 2A) and squamous-papillary (Fig. 2B) craniopharyngioma samples were not stained by TUNEL-POD, indicating a low rate of apoptosis.

**Figure 3:**
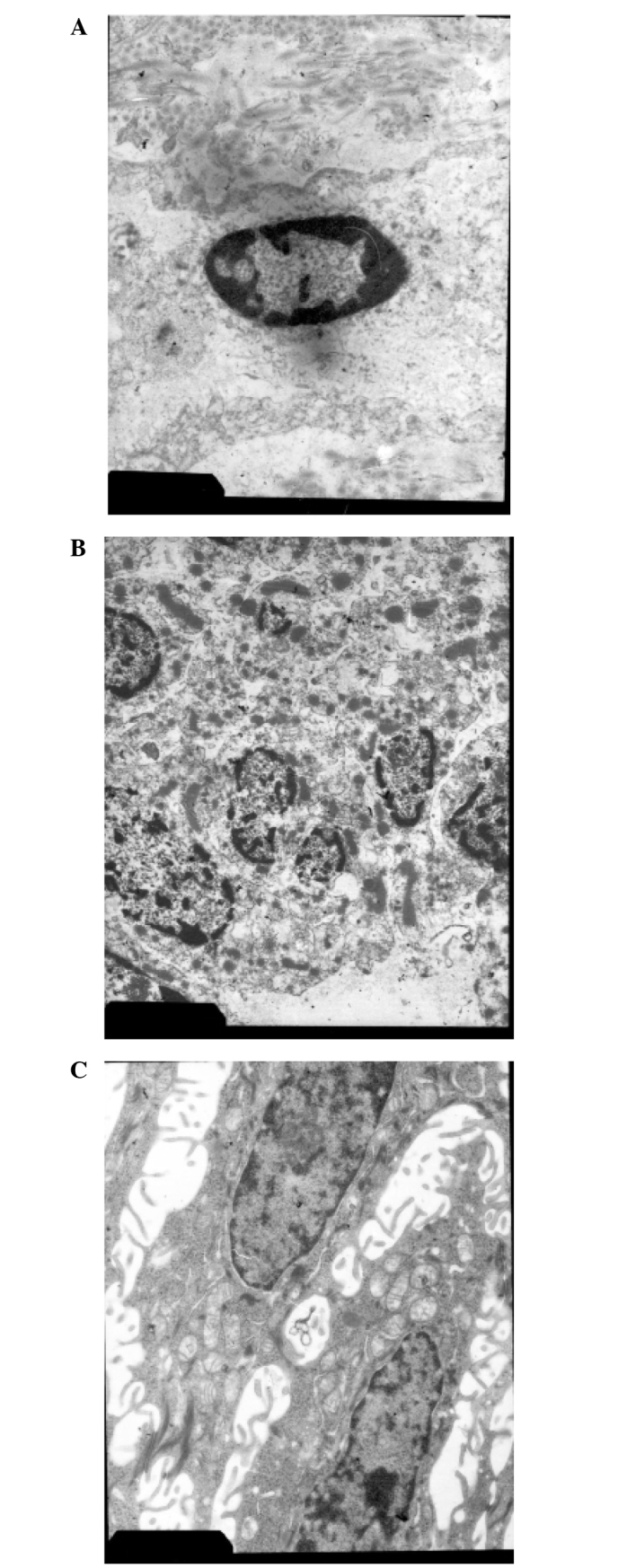
Electron microscopy of various types of tumor cell, including (A) apoptotic, (B) necrotic and (C) craniopharyngioma tumor cells (magnification, ×10,000).

Different types of tumor cells were observed using electron microscopy. The nuclei of the apoptotic tumor cells (Fig. 3A) were oval in shape and condensed into a uniformly dense structure. The chromatin had migrated to the edge of the nucleus on the inner side of the nuclear membrane, and the nuclear and cell membranes were integrated. Necrotic tumor cells appeared to die in clusters with damaged nuclear membranes and irregularly broken and condensed chromatin, forming sparse reticular clots (Fig. 3B). The ultrastructure of the craniopharyngioma cells was similar to that of squamous epithelial cells (Fig. 3C). The cells were densely packed into stratified epithelial clumps with a large, oval-shaped and slightly deformed nucleus, and an integrated, but rugged nuclear membrane. Chromatin distribution was even, abundant tonofibrils were observed in the cytoplasm, and mitochondria, rough endoplasmic reticulum, ribosomes and other organelles were clearly visible. Furthermore, abundant microvilli were observed on the cell surface, and the cells were loosely arranged with a vast cell gap in which integrated intercellular desmosomes and multiple connections appeared to have formed.

### Survivin protein expression levels

**Figure 4:**
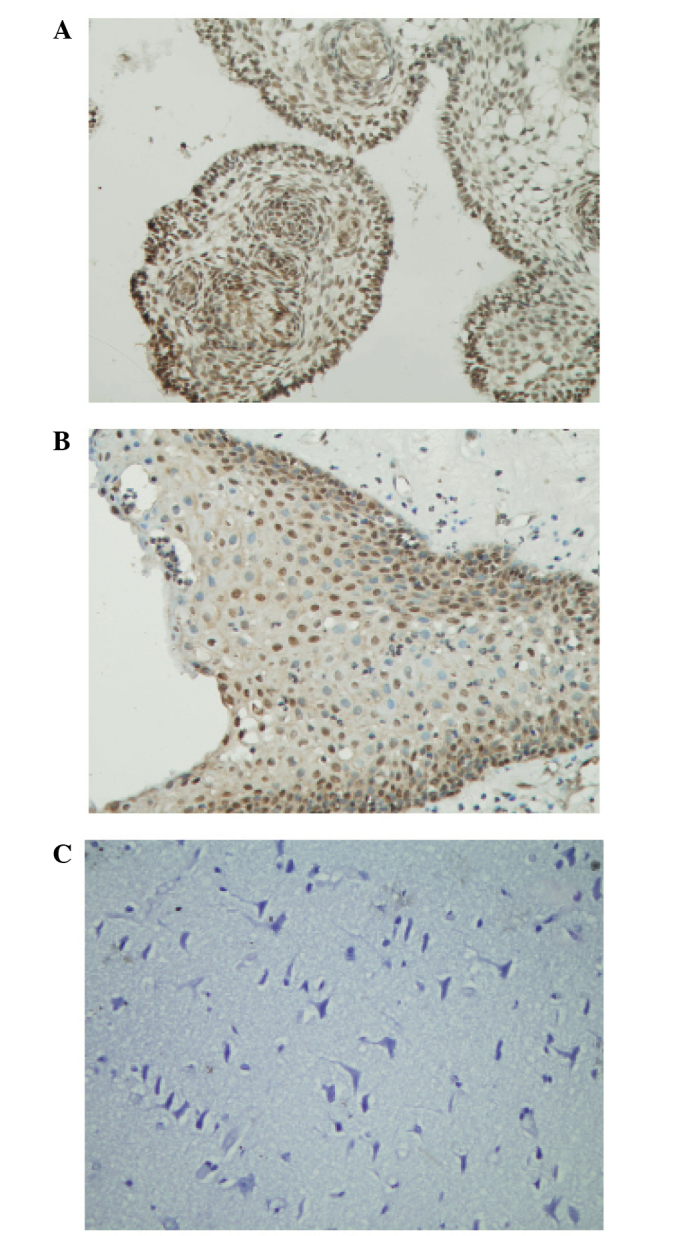
Survivin expression levels in 50 craniopharyngioma samples, including (A) adamantinomatous and (B) squamous-papillary craniopharyngioma tissues, and (C) 10 healthy control samples (magnification, ×400).

Survivin protein expression was detected in all 50 craniopharyngioma samples in the cytoplasm or the nucleus. In adamantinomatous craniopharyngioma (Fig. 4A), significant survivin expression was detected in the majority of tissue sections and the cells predominantly exhibited higher expression levels in the nucleus compared with the cytoplasm. High survivin expression was predominantly observed in the palisade-like epithelial cells, as well as cells in the finger-like or clump-like cell clusters. The stellate reticular cells exhibited relatively weak survivin expression levels and the expression pattern in the squamous-papillary craniopharyngioma (Fig. 4B) was similar to that of the adamantinomatous craniopharyngioma. Furthermore, survivin-positive cells were predominantly terminally differentiated squamous epithelial cells. Survivin was highly expressed in the nucleus and poorly expressed in the cytoplasm of the craniopharyngioma cells; however, no survivin expression was detected in the 10 healthy brain tissue sections (P<0.001; Fig. 4C).

The images of Fig. 4A, B and C were analyzed using Image-Pro Plus software, and the IOD was selected as the parameter to indicate survivin protein expression level, with a higher IOD indicating higher survivin expression. Survivin expression was found to be significantly higher in the craniopharyngioma tissues (50 cases) compared with the healthy control (10 cases) samples (P<0.001). All patients ≤16 years old exhibited adamantinomatous craniopharyngioma, while among the 33 patients >16 years old, 16 exhibited the adamantinomatous tumor type (48.5%) and 17 patients exhibited the squamous-papillary type (51.5%). Furthermore, survivin expression was significantly higher in adamantinomatous craniopharyngioma (33 cases) compared with squamous-papillary (17 cases) craniopharyngioma (P=0.036). Compared with the female craniopharyngioma patients (18 cases), the level of survivin expression was significantly higher in the overall male craniopharyngioma patient population (32 cases) (P=0.002) and in those males with the adamantinomatous tumor type (P<0.001). However, the difference in survivin expression levels between the male and female squamous-papillary craniopharyngioma patients was not statistically significant. Survivin expression was significantly higher in the patients ≤16 years old (17 cases) compared with the patients >16 years old (33 cases) (P=0.002). No statistically significant differences were detected between the three subtypes of craniopharyngioma (cystic, 26 cases; solid, 13 cases; and mixed-type, 11 cases); or between the different disease courses (≤12-month disease course, 39 cases; and >12-month disease course, 11 cases). However, the survivin expression levels were significantly higher in the recurrent craniopharyngioma tumors (9 cases) compared with the non-recurrent tumors (41 cases) (P=0.011).

### Regression analysis

All the patients in the present study were followed up by phone until the end of November 2010; however, the required information was only collected from 46 patients. The cases of craniopharyngioma were divided into five grades: Grades I, II, III and IV, as described by Carmel *et al* (17), plus mortality. Multinomial and ordinal logistic regression was performed to evaluate the candidate risk factors for prognosis. In the present model, prognosis was defined as a dependent variable, and pathological type, age, tumor subtype, complications and survivin expression level (IOD value) were defined as independent variables.

A significant correlation was demonstrated between complication as a risk factor for the prognosis of craniopharyngioma patients and prognosis (P<0.05); patients with no complications exhibited a significantly more favorable prognosis compared with patients who experienced complications. However, no significant correlation was detected among all other candidate risk factors.

## Discussion

Controversy regarding the origin of craniopharyngioma has always been present, and two expounding theories currently exist: The embryonic origin theory and the metaplasia theory. Certain individuals believe that it is the residual ectoderm cells in the primary cranial pharyngeal tube and anterior pituitary that form craniopharyngioma (18,19). In the present study, it was identified that all patients ≤16 years old exhibited adamantinomatous craniopharyngioma. We speculate that adamantinomatous craniopharyngioma may originate from residual ectoderm cells, which appears to support Erdheim’s embryonic origin theory (20). However, it is unlikely that squamous-papillary craniopharyngioma also originates from residual embryonic ectoderm cells, since the present study identified no cases of squamous-papillary craniopharyngioma in patients ≤16 years old. Instead, it was proposed that squamous-papillary craniopharyngioma may have a different origin. The metaplasia theory states that craniopharyngioma originates from metaplasia of adenohypophysis and the anterior infundibular squamous epithelium (2,14,21). In the present study, it was identified that no individuals ≤16 years old exhibited squamous-papillary craniopharyngioma, contradicting the embryonic origin theory, in which such a cell nest should be primarily be observed in children. In addition, such cell nests are commonly observed in patients who are >20 years old (22). However, the present study identified that survivin is expressed in significantly higher levels in adamantinomatous craniopharyngioma tissues compared with squamous-papillary craniopharyngioma tissues. Under normal circumstances, survivin is only expressed during embryonic and fetal development, and not in adults, thus, it was speculated that adamantinomatous craniopharyngioma originates from residue embryo tissues, while squamous-papillary craniopharyngioma originates from squamous metaplasia.

Histologically, craniopharyngioma can be divided into the adamantinomatous and squamous-papillary types. The majority of children with craniopharyngioma exhibit the adamantinomatous form, however, an equal proportion of the two types are observed in adults (2). Furthermore, Tavangar *et al* (23) identified that squamous-papillary craniopharyngioma only occurs in adults; this form of the disease has fewer clinical manifestations and a lower risk of recurrence compared with adamantinomatous craniopharyngioma. Additionally, Crotty *et al* (24) identified that adamantinomatous craniopharyngioma predominantly occurs in adolescents and was more invasive than squamous-papillary craniopharyngioma. Consistent with these previous studies, the present study observed that all the patients who were ≤16 years old exhibited adamantinomatous craniopharyngioma and that all the patients who were >16 years old had an equal chance of developing each type of craniopharyngioma. A low recurrence rate was observed (9/50 cases; 18%), in accordance with the benign nature of adamantinomatous craniopharyngioma. Of the nine cases of recurrence, two (22.2%) were of a squamous-papillary type and seven (77.8%) were adamantinomatous, consistent with the above mentioned studies. Light microscopy demonstrated that adamantinomatous craniopharyngioma often invaded the surrounding healthy tissue, however, this phenomenon was not observed in the squamous-papillary craniopharyngioma tissues, thus, adamantinomatous craniopharyngioma appeared to exhibit a higher level of invasiveness. However, electron microscopy of 10 cases of craniopharyngioma by Kawano *et al* (25) demonstrated that although the microscopic morphology of the two types of craniopharyngioma were significantly different, their ultrastructure was largely similar. In the present study, abundant mitochondria were identified in the craniopharyngioma cells, indicating active metabolism, and well-developed rough endoplasmic reticulum and ribosomes were clearly visible, which is typical of fully differentiated and actively functioning cells. Despite the benign clinical manifestation and biological behavior of craniopharyngioma observed in the present study, these features appear to oppose the traditionally recognized benign nature of craniopharyngioma. Furthermore, the ultrastructure of craniopharyngioma appears to exhibit malignant potential. A high ratio of euchromatin, a large number of nuclear pores and large nucleoli were observed in the nucleus of the craniopharyngioma cells, indicating a high level of protein synthesis. Therefore, craniopharyngioma may not be benign as previously hypothesized. In addition, electron microscopy identified that craniopharyngioma features abundant tonofibrils and microvilli; the latter is associated with the solidity and material transport, indicating solidity of craniopharyngioma and active cell contraction, including movement, cytoplasm flow, phagocytosis and excretion.

In 1997, survivin, a novel inhibitor of apoptosis, was identified (26). A high level of survivin expression was found in all common human malignancies at the mRNA and protein levels. Hassounah *et al* (27) evaluated survivin expression levels in various benign neurological tumors and the results indicated that survivin expression may be an early event in tumorigenesis, providing a favorable condition for the development of malignant or benign tumors. In the present study, it was identified that survivin protein is expressed at varying levels in craniopharyngioma, however, it is not expressed in healthy brain tissue. It is proposed that survivin expression levels in craniopharyngioma may exhibit anti-apoptotic effects. In addition, it was identified that survivin expression levels are significantly different in different pathological types, genders and age groups, and between recurrent and non-recurrent craniopharyngioma patients. The present study provided novel data regarding survivin expression levels in neurological benign tumors, highlighting the importance of survivin in the occurrence and development of brain tumors. In addition, survivin is expressed at higher levels in adamantinomatous craniopharyngioma compared with squamous-papillary craniopharyngioma, which may partially explain the higher recurrence rate of the adamantinomatous tumor type. Furthermore, it was identified that males have higher ratio of adamantinomatous to squamous-papillary craniopharyngioma compared with females. Among the adamantinomatous craniopharyngioma patients, survivin expression levels were higher in the males compared with the females, while male and female squamous-papillary craniopharyngioma patients demonstrated no significant difference in survivin expression. The higher proportion of male adamantinomatous craniopharyngioma patients and the higher survivin expression levels in male adamantinomatous craniopharyngioma patients may have resulted in the typically high survivin expression levels observed overall in males compared with females. The results of the present study indicate that survivin may exhibit a stronger anti-apoptotic affect in male patients compared with female patients, indicating a higher invasiveness of craniopharyngioma in males, however, additional clinical observation is required to clarify this hypothesis. Furthermore, it was identified that survivin protein expression levels are higher in recurrent craniopharyngioma cases compared with non-recurrent cases, indicating that higher survivin expression levels may inhibit apoptosis and promote tumor recurrence.

In conclusion, the current study indicates that survivin may regulate the cell cycle and apoptosis in the tumorigenesis of craniopharyngioma. Survivin may significantly inhibit the apoptosis of the tumor cells, thus increasing the aggressiveness of benign tumors and the malignancy of malignant tumors, as well as increasing the likelihood of tumor recurrence. Therefore, survivin may be useful as a predictor of craniopharyngioma patient prognosis, however, additional studies on larger patient samples are required to confirm this association.
